# Structural and electrical properties of catalyst-free Si-doped InAs nanowires formed on Si(111)

**DOI:** 10.1038/srep16652

**Published:** 2015-11-19

**Authors:** Dong Woo Park, Seong Gi Jeon, Cheul-Ro Lee, Sang Jun Lee, Jae Yong Song, Jun Oh Kim, Sam Kyu Noh, Jae-Young Leem, Jin Soo Kim

**Affiliations:** 1 Division of Advanced Materials Engineering & Research Center of Advanced Materials Development, Chonbuk National University, Jeonju 561-756, Republic of Korea; 2 Department of Materials Science and Engineering, Korea Advanced Institute of Science and Technology, Daejeon 305-338, Republic of Korea; 3 Materials Genome Center, Korea Research Institute of Standards and Science, Daejeon 305-340, Republic of Korea; 4 School of Nano Engineering, Inje University, Gimhae 621-749, Republic of Korea

## Abstract

We report structural and electrical properties of catalyst-free Si-doped InAs nanowires (NWs) formed on Si(111) substrates. The average diameter of Si-doped InAs NWs was almost similar to that of undoped NWs with a slight increase in height. In the previous works, the shape and size of InAs NWs formed on metallic catalysts or patterned structures were significantly changed by introducing dopants. Even though the external shape and size of the Si-doped NWs in this work were not changed, crystal structures inside the NWs were significantly changed. For the undoped InAs NWs, both zincblende (ZB) and wurzite (WZ) structures were observed in transmission-electron microscope images, where the portion of WZ structure was estimated to be more than 30%. However, only ZB was observed with an increase in stacking fault (SF) for the Si-doped NWs. The undoped and Si-doped InAs NWs were used as channels of four-point electrical measurements with Al/Ni electrodes to investigate electrical properties. The resistivity calculated from the current-voltage curve of a Si-doped InAs NW showed 1.32 × 10^−3^ Ωcm, which was dramatically decreased from 10.14 × 10^−3^ Ωcm for the undoped InAs NW. A relatively low resistivity of catalyst-free Si-doped InAs NWs was achieved without significant change in structural dimensions.

In the past several years, semiconductor nanowires (NWs) including GaAs/Si and InAs/Si have been actively studied in terms of fundamental physics and potential applications to opto-electronic devices due to their unique and superior electrics, thermoelectrics and optics properties^1^,^2^,^3^,^4^,^5^,^6^. Most of the InAs NWs on Si substrates have been fabricated by using metallic catalysts such as Au and Ni^7^,^8^,^9^. However, metallic catalysts may create deep levels in the energy bandgap mainly due to chemical contamination. This may potentially degrade the electrical and optical properties of InAs NWs. Recently, several research groups reported catalyst-free InAs NWs using Volmer-Weber three-dimensional (3D) islands and the vapour-liquid-solid (VLS) growth method^10^,^11^,^12^. In the Volmer-Weber growth mode, interactions between adatoms are stronger than those between the adatom and surface, leading to the formation of InAs clusters or islands on Si substrates. Many research groups reported doping effects on the electrical, structural and optical properties of InAs NWs for the application of optoelectronic devices^13^,^14^,^15^,^16^,^17^,^18^,^19^,^20^. Most of the previous reports on doping for InAs NWs showed a significant change in size and shape^15^,^16^,^17^,^18^,^19^. E. Dimakis *et al.* demonstrated that the increase in Si-doping level led to a decrease in height and increase in diameter compared to that of undoped InAs NWs. Also, they demonstrated that the increase in the Si-doping level resulted in the decrease of the WZ/ZB ratio from an x-ray diffraction reciprocal-space mapping study^18^. As a result, the precise comparison of electrical properties between undoped and doped-InAs NWs has been limited. H. Ghoneim *et al.* demonstrated *in*-*situ* doping to InAs NWs using diethyl telluride, disilane (Si_2_H_6_), hydrogen sulfide (H_2_S), and carbon tetrabromide inside metal-organic vapour phase epitaxy (MOVPE). Introducing low-level Si_2_H_6_ and H_2_S to InAs NWs showed the same growth rate and morphology as those of undoped NWs. However, the axial and radial dimensions were significantly changed at relatively high doping condition. In addition, for the Si-doped InAs NWs, the low resistivity of 0.26 × 10^−3^ Ωcm was achieved^19^. C. Thelander *et al.* demonstrated the minimum resistivity to 2.1 × 10^−3^, 2.8 × 10^−3^, 1.9 × 10^−3^, 0.23 × 10^−3^, and 1 × 10^−3^ Ωcm for the n-type InAs NWs doped using silicon tetrabromide (chemical beam epitaxy (CBE)), ditertiarybutylselenium (DTBSe) (CBE), H_2_S (MOVPE), DTBSe (MOVPE), and tetraethyltin (MOVPE), respectively^20^. Wirths *et al.* presented a transmission-electron microscope (TEM) study on InAs NWs formed on patterned structures (MOVPE) with respect to doping levels, where the irregular crystal characteristics of InAs NWs were shown^15^. According to the previous results, introducing doping led to the drastic modification in size and shape of InAs NWs. Moreover, most of the results were obtained from InAs NWs fabricated on metallic catalysts or patterned structures. That is, the effect of doping on structural and electrical properties including reduction in resistivity of catalyst-free InAs NWs has not yet been reported.

In this paper, we report the effects of Si-doping on the structural and electrical properties of catalyst-free InAs NWs formed on Si(111). For Si-doped InAs NWs, the external structural dimensions including diameter and density were not modified compared to those of undoped NWs. To investigate the electrical properties of Si-doped InAs NWs, current-voltage (I–V) curves obtained from four-point electrical measurements were estimated. The resistivity of a Si-doped InAs NW was calculated to be 1.32 × 10^−3^ Ωcm, which was effectively reduced from 10.14 × 10^−3^ Ωcm for the undoped InAs NW.

## Results

**Figure 1:**
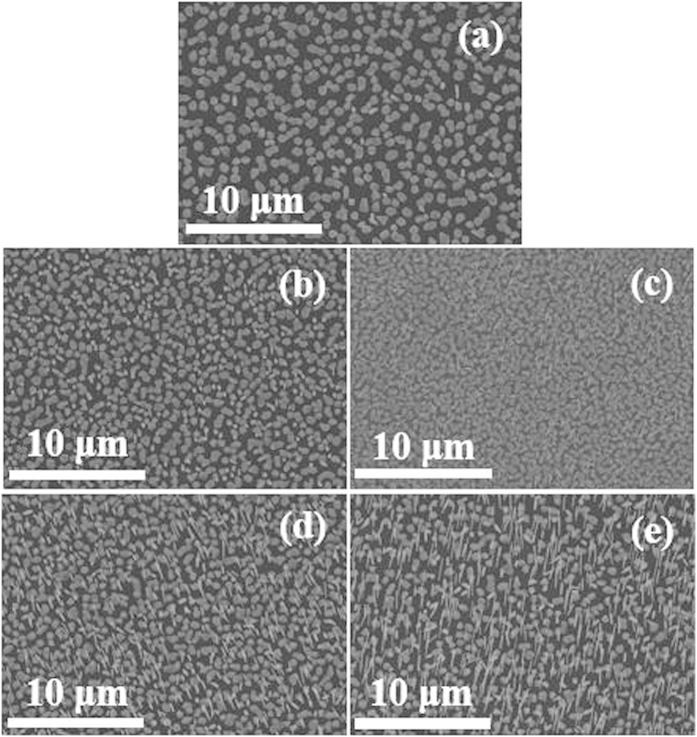
FE-SEM images of (**a**) UN50, (**b**) UN100, (**c**) UN200, (**d**) UN300, and (**e**) UN400.

**Figure 2:**
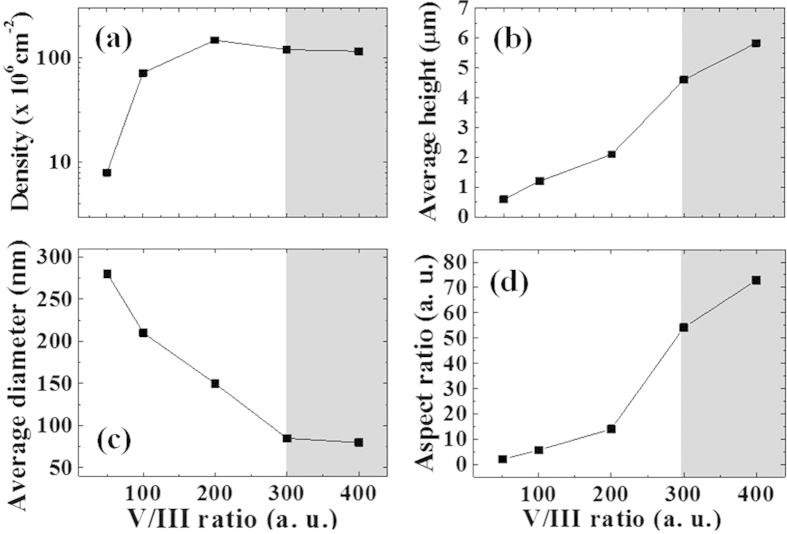
Summary of spatial density, average heights, average diameter, and aspects ratio with V/III ratio.

Figure 1(a) shows plan-view FE-SEM images of InAs NW samples grown at different V/III ratios ranging from 50 to 400. Figure 2(a–d) show the summaries on the spatial density, average height, average diameter, and aspects ratio (height/diameter), respectively, of InAs NWs with respect to the V/III ratios. The spatial density of InAs NWs on Si(111) was increased from 8 × 10^6^ to 1.5 × 10^8^ cm^−2^ with increasing V/III ratio from 50 to 200. With further increasing V/III ratio, the density of InAs NWs was not increased. The average heights of InAs NWs were measured to be 0.6, 1.2, 2.1, 4.6, and 5.8 μm for the V/III ratios of 50, 100, 200, 300, and 400, respectively. The average diameters of InAs NWs were 280, 210, 150, 85, and 80 nm for the UN50, UN100, UN200, UN300, and UN400 samples, respectively. The aspect ratios of InAs NWs were calculated to be 2.1, 5.7, 14, 54, and 72 for the V/III ratios of 50, 100, 200, 300, and 400, respectively. Because the large amount of As adatoms at the relatively high V/III ratio can crystallize more In to the InAs structures, the relatively high V/III ratio can lead to high density of the NWs.

**Figure 3:**
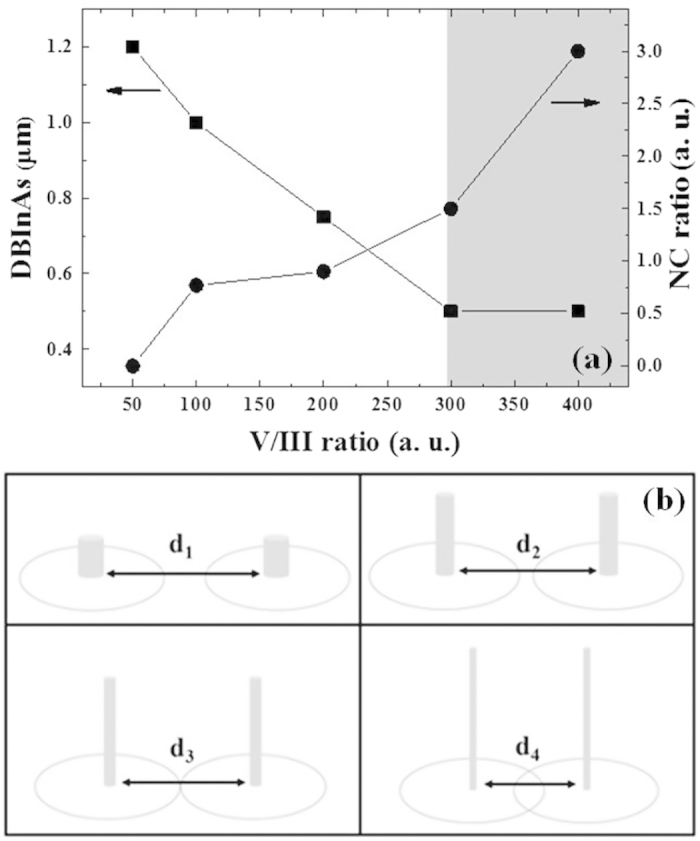
(**a**) DBInAs and NC ratio with the V/III ratio, (**b**) schematic illustration for the formation of InAs NWs with respect to the DBInAs (d_1_ > d_2_ > d_3_ > d_4_) according to the V/III ratios.

Figure 3(a) shows the distances between the InAs structures (DBInAs) and the ratios of InAs NWs to chunks (NC Ratio) with respect to the V/III ratio. The DBInAs varied from 1.2 to 0.5 μm with increasing the V/III ratios. For relatively short DBInAs for the UN300 and UN400 samples, the probability of InAs nucleation can be increased, resulting in the increase in spatial density of NWs. In addition, the dense InAs NWs limit the migration length of In adatoms, leading to the formation of relatively long NWs compared to those of the long DBInAs. Since there is relatively high probability for In adatoms to crystallize to InAs under high As condition, the interaction between In atoms and Si surface is surely reduced^12^,^21^. As a result, the NC ratio was increased geometrically from 0.8 to 3 with increasing V/III ratio. Figure 3(b) shows a schematic illustration of the growth variety of InAs NWs with the DBInAs (d_1_ > d_2_ > d_3_ > d_4_) according to the V/III ratio. The nucleation of the InAs structures was mostly affected by the migration length of In adatoms influenced by the amount of As flux. That is, relatively high As flux can result in the formation of dense and higher InAs NWs.

**Figure 4:**
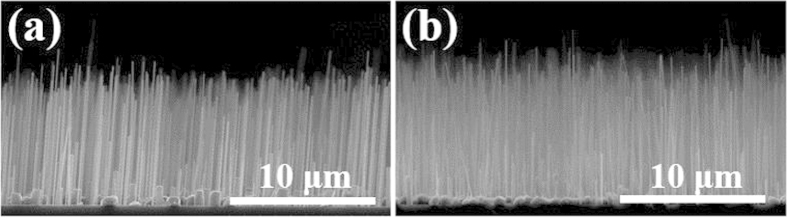
FE-SEM images of (**a**) undoped and (**b**) Si-doped InAs NWs.

Figure 4 shows cross-sectional FE-SEM images of undoped and Si-doped InAs NWs formed at the V/III ratio of 400. Si-doped InAs NWs were formed at the same growth conditions for the undoped NWs except Si-doping. The spatial density and diameter of Si-doped InAs NWs were similar to those of undoped NWs, and the average height was slightly increased. That is, there is no significant change in the external structure and dimensions of InAs NWs by doping. In the previous report, when Si was doped into catalyst-free InAs NWs, the average diameter was significantly increased from 60 to 80 nm with the reduction in height compared to that of the undoped NWs^18^, because higher Si flux induced a larger nucleus and slower axial growth. In addition, if the number of Si atoms was greater than the critical amount, the diffusion length of the In adatoms on the surface was reduced, resulting in the formation of relatively large NWs with reduced heights^19^.

**Figure 5:**
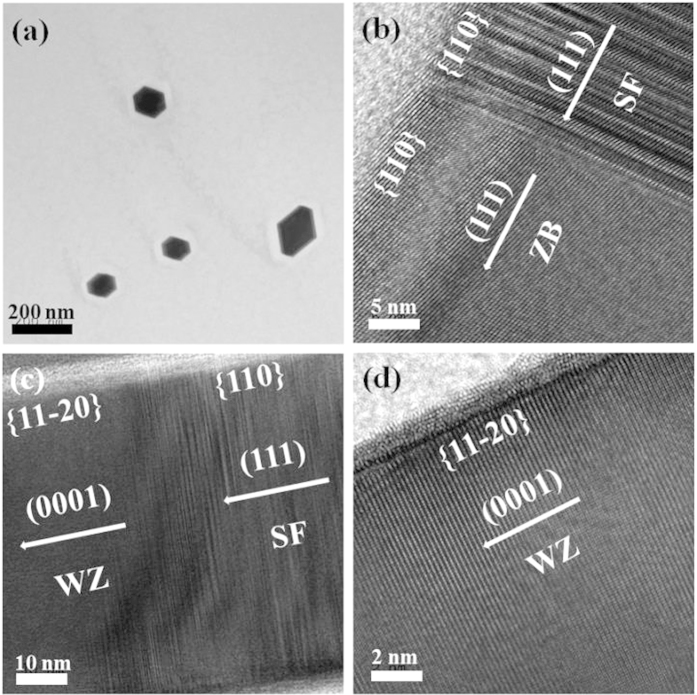
(**a**) plan-view TEM image of undoped InAs NWs with the different hexagonal shapes. High-resolution TEM images for the (**b**) region exhibiting ZB and SF, (**c**) region exhibiting WZ and SF, and (**d**) region exhibiting WZ only.

Figure 5 shows TEM images of undoped InAs NWs. Figure 5(a) reveals the different hexagonal shapes of InAs NWs. In the VLS growth mode, the nucleation usually appeared on various sites of the substrate, because catalysts and patterns were not used. Figure 5(b) shows the high-resolution TEM image for the region exhibiting both ZB and SF, in which the growth direction and side facet are (111) and {110}, respectively. Figure 5(c) is the TEM image for the region with WZ and SF. Because the crystal structure was changed from SF to WZ, the growth direction and side facet were consequently changed to (0001) and {11–20}, respectively. The WZ structure shown in Fig. 5(d) was mostly observed at the upper region of an undoped InAs NW.

**Figure 6:**
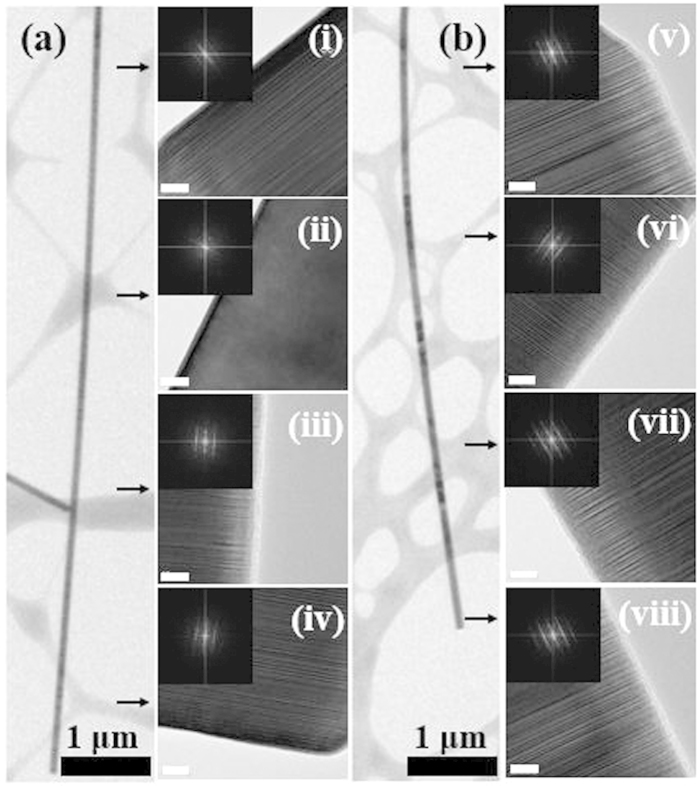
TEM images of (**a**) undoped InAs NWs and (**b**) Si-doped InAs NWs, and diffraction patterns with HR TEM images (scale bar: 10 nm).

Figure 6(a,b) show TEM images with the insets of diffraction patterns for different positions of undoped and Si-doped InAs NWs, respectively. In Fig. 6(a), the crystal structure at positions (i), (iii), and (iv) shows ZB. Also, a pure WZ structure was observed at position (ii) of the undoped NW. The portion of WZ structure for the undoped NW was estimated to be over 30%. At the bottom of the InAs NW on Si, the diameter is relatively thin with high strain field. However, only a ZB structure without WZ was observed for the Si-doped InAs NW as shown in Fig. 6(b). For the positions (v), (vi), (vii), and (viii), the diffraction patterns clearly show the ZB structure. In addition, the amount of SF for the Si-doped NW was relatively larger than that of the undoped InAs NW. This is related to the substitution of Si in an InAs structure such as Si_In_ and Si_As_. Also, Si can be located at native vacancy sites. These defects still maintain strain, which can block the conversion of the crystal structure to WZ. In FE-SEM images of Fig. 4, the shapes and sizes of Si-doped InAs NWs were not significantly changed, which is quite different from the previous results that the structural dimension of doped NWs were changed. This result can be explained by the modification in the internal crystal structure of InAs NWs from WZ to ZB by doping Si as shown in TEM images of Fig. 6(b). The increase in the portion of ZB structure for a Si-doped InAs NW can be explained by a concept of kinetic phase diagram separating the domains of WZ and ZB as a function of difference in liquid–solid step free energy between a ZB and a WZ nucleus^22^,^23^,^24^,^25^. Algra *et al.* reported that the increase in the formation rate of ZB-structured InP NWs, formed on colloidal gold particles, by adding zinc dopant was most likely to the reduction in the liquid–solid step energy of the ZB structure with respect to the WZ structure^25^. Similarly, the formation of ZB-type Si-doped InAs NWs in this work is mostly attributed to the reduction in the liquid–solid step free energy of the ZB structure by introducing Si atoms.

**Figure 7:**
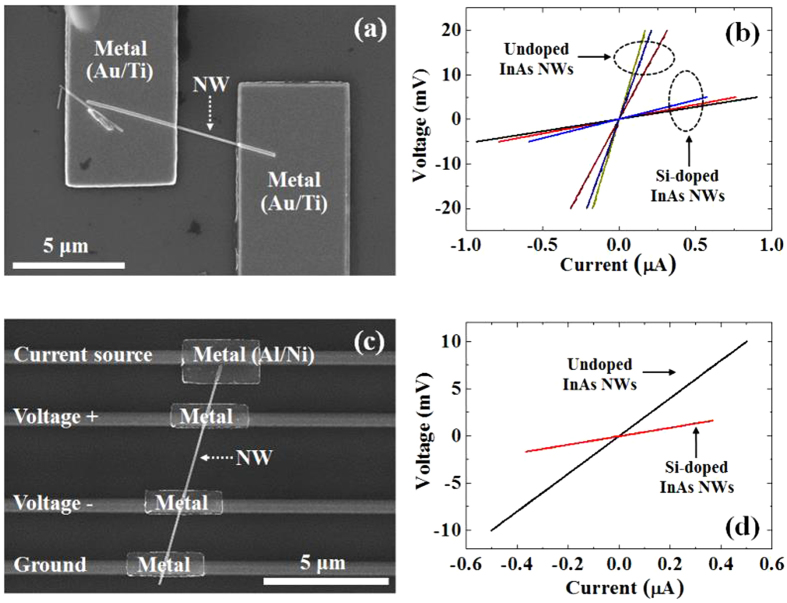
(**a**) FE-SEM image of an InAs NW with two-point metal contacts and (**b**) I–V curves. (**c**) FE-SEM image of an InAs NW with four-point metal configuration and (**d**) I–V curves.

Figure 7(a) shows an FE-SEM image of an InAs NW with metal contacts (denoted as relatively white squares) for two-point probe characterization, where the distance between metal contacts is 3 μm. The diameter of the InAs NW was measured to be 85 nm. In order to use a less-defective region, the center areas of NWs were chosen as channels. In Fig. 7(b), the two-point I-V curves for undoped and Si-doped InAs NWs are shown, where the three different sets of each NW device were evaluated to obtain more precise and statistical information. Since the shape of InAs NWs differed as shown in the TEM image of Fig. 5(a), the electrical properties may also differ, depending on the shape of the NWs. The resistivity of the three different sets of devices with an undoped-InAs NW as a channel was measured to be 8.73 × 10^−3^, 13.16 × 10^−3^, and 16.49 × 10^−3^ Ωcm, respectively. Meanwhile, the resistivity for the Si-doped InAs NWs was measured to be 0.76 × 10^−3^, 0.90 × 10^−3^, and 1.19 × 10^−3^ Ωcm, respectively, showing a significant reduction compared to those of the undoped InAs NWs. Usually, resistivity strongly depends on the InAs NW itself, the contact-area treatment, and contact metal electrodes. To rule out contribution of a varying contact resistance, the measurements were performed in a four-point metal configuration shown in Fig. 7(c), where the distance between electrical contacts was measured to be 2.6 μm. The resistivity of the devices estimated from the I–V curves shown in Fig. 7(d) was 10.14 × 10^−3^ and 1.32 × 10^−3^ Ωcm for an undoped and Si-doped InAs NWs, respectively. The resistivity for the Si-doped InAs NW obtained from the four-point electrical measurements was also drastically reduced from that of the undoped NW, which is similar to the results from two-point contact measurements. This is related that the realization of Ohmic contact to InAs is less sensitive to dopant type, doping level, and conditions compared to other III–V semiconductors. For InAs, it has been observed that the surface Fermi level is pinned in the conduction band regardless of the crystal orientation or dopant type, which makes it relatively easy to realize Ohmic contact to InAs. However, this pinning makes it difficult to achieve p-type conductivity in InAs thin film and NWs resulting in the n-type conductivity for most of intrinsic and doped InAs NWs^26^,^27^,^28^,^29^. From this point of view, the significant reduction in resistivity is largely related to the change in the characteristics of InAs NW itself. In addition, we consider the influence of the increase in SF on the resistivity for a Si-doped InAs NW. The increased stacking faults may degrade electron mobility resulting in the increase in resistivity of InAs NWs. However, the resistivity for the Si-doped InAs NW in our work was effectively decreased. This result can be explained by the fact that the large effective carriers provided from high doping level of 1 × 10^19^ cm^−3^ overcompensates for the degradation in carrier mobility due to sacking fault. In the previous report on transport properties of InAs NW with high stacking fault, the carrier mobility was increased with increasing Si-doping level^15^. Although the reduction in the resistivity of InAs NWs by the introduction of Si has been recently reported, the structural dimensions of InAs NWs were considerably changed^15^,^16^,^17^,^18^,^19^. However, a relatively low resistivity of Si-doped InAs NWs in this work was achieved without significant change in structural dimensions. In addition, this is the first observation on the improvement in electrical properties of catalyst-free InAs NWs.

## Discussion

We reported the structural and electrical properties of Si-doped InAs NWs by comparing undoped InAs NWs. By controlling the V/III ratio of InAs, the various densities, diameters, and heights of NWs were obtained. The average diameter of InAs NWs was not modified by Si doping. Instead, the interior crystal structure was significantly changed by doping. That is, only the ZB structure was observed with an increase in the SF. The resistivity calculated from the I–V curves of Si-doped InAs NWs showed 1.32 × 10^−3^ Ωcm, which is significantly reduced from 10.14 × 10^−3^ Ωcm for the undoped InAs NWs. The improvement in electrical conductivity for the Si-doped NWs is mostly related that the large effective carriers provided from high doping level overcompensates for the degradation in carrier mobility due to sacking fault.

## Methods

The InAs NWs were grown on Si(111) substrates using a Riber32P molecular-beam epitaxy (MBE) with solid sources. Before depositing InAs NWs, Si(111) substrates were chemically cleaned using a standard wet-etching process with acetone, methanol, isopropyl alcohol, and deionized water (DI-water). A deoxidation process was then performed using hydrofluoric acid (HF) solution [HF (1) + DI-water (20)]. After the chemical deoxidation process, Si(111) substrates were immediately loaded into a load-lock chamber of an MBE system. The growth rate of InAs NWs was 0.01 Å/second, estimated from nominal two-dimensional (2D) epitaxial growth. The growth temperature for InAs NWs was fixed at 430 °C. The V/III ratios for the formation of NWs were 50 (UN50 sample), 100 (UN100 sample), 200 (UN200 sample), 300 (UN300 sample), and 400 (UN400 sample), which were obtained by controlling the arsenic (As) flux at a fixed indium (In) beam-equivalent pressure of 3 × 10^−8^ torr, as shown in Table 1.

For the growth of Si-doped InAs NWs, Si with the nominal doping level of 1 × 10^19^ cm^−3^ measured at 2D growth was simultaneously supplied during the deposition period of InAs under the V/III ratio of 400. The external dimensions of InAs NWs were measured using a field-emission scanning-electron microscope (FE-SEM) with a Hitachi S-4800 model. The internal structural properties of InAs NWs were measured using a transmission electron microscope (TEM, model of FEI tecnai G^2^ F30 S-Twin). InAs NWs were separated from Si substrates using ultrasonic cleaning system in ethanol for TEM measurements. To investigate the electrical properties, InAs NWs separated from Si substrates were moved onto a SiOx substrate using a tungsten tip. Since van der Waals force acted between the InAs NWs and the tungsten tip, the NWs were easily handled and transferred onto the SiOx substrate. Electric contacts at both ends of the InAs NWs for two-point measurements were then realized using e-beam lithography and sputter with Au (100 nm) and Ti (10 nm) on the SiOx substrate. Also, the Al (100 nm) and Ni (15 nm) metals were used for the fabrication of four-point electrical configuration on the SiOx substrate.

## Additional Information

**How to cite this article**: Park, D. W. *et al.* Structural and electrical properties of catalyst-free Si-doped InAs nanowires formed on Si(111). *Sci. Rep.* **5**, 16652; doi: 10.1038/srep16652 (2015).

## Figures and Tables

**Table 1:** Details for the growth of InAs NWs.

Sample	In flux (torr)	As flux (torr)	V/III ratio(arb. units)
UN 50	3 × 10^−8^	1.5 × 10^−6^	50
UN 100	3 × 10^−8^	3.0 × 10^−6^	100
UN 200	3 × 10^−8^	6.0 × 10^−6^	200
UN 300	3 × 10^−8^	9.0 × 10^−6^	300
UN 400	3 × 10^−8^	1.2 × 10^−5^	400
